# Retention and Loss of RNA Interference Pathways in Trypanosomatid Protozoans

**DOI:** 10.1371/journal.ppat.1001161

**Published:** 2010-10-28

**Authors:** Lon-Fye Lye, Katherine Owens, Huafang Shi, Silvane M. F. Murta, Ana Carolina Vieira, Salvatore J. Turco, Christian Tschudi, Elisabetta Ullu, Stephen M. Beverley

**Affiliations:** 1 Department of Molecular Microbiology, Washington University School of Medicine, St. Louis, Missouri, United States of America; 2 Department of Internal Medicine, Yale University Medical School, New Haven, Connecticut, United States of America; 3 Department of Epidemiology & Public Health, Yale University Medical School, New Haven, Connecticut, United States of America; 4 Department of Cell Biology, Yale University Medical School, New Haven, Connecticut, United States of America; 5 Department of Biochemistry, University of Kentucky Medical Center, Lexington, Kentucky, United States of America; Oregon Health & Science University, United States of America

## Abstract

RNA interference (RNAi) pathways are widespread in metaozoans but the genes required show variable occurrence or activity in eukaryotic microbes, including many pathogens. While some *Leishmania* lack RNAi activity and *Argonaute* or *Dicer* genes, we show that *Leishmania braziliensis* and other species within the *Leishmania* subgenus *Viannia* elaborate active RNAi machinery. Strong attenuation of expression from a variety of reporter and endogenous genes was seen. As expected, RNAi knockdowns of the sole *Argonaute* gene implicated this protein in RNAi. The potential for functional genetics was established by testing RNAi knockdown lines lacking the paraflagellar rod, a key component of the parasite flagellum. This sets the stage for the systematic manipulation of gene expression through RNAi in these predominantly diploid asexual organisms, and may also allow selective RNAi-based chemotherapy. Functional evolutionary surveys of RNAi genes established that RNAi activity was lost after the separation of the *Leishmania* subgenus *Viannia* from the remaining *Leishmania* species, a divergence associated with profound changes in the parasite infectious cycle and virulence. The genus *Leishmania* therefore offers an accessible system for testing hypothesis about forces that may select for the loss of RNAi during evolution, such as invasion by viruses, changes in genome plasticity mediated by transposable elements and gene amplification (including those mediating drug resistance), and/or alterations in parasite virulence.

## Author Summary

RNAi interference pathways play fundamental roles in eukaryotes and provide important methods for the analysis of gene function. Occasionally RNAi has been lost, precluding its use as a tool, as well as raising the question of what forces could lead to loss of such a key pathway. Genomic and functional studies previously showed that within trypanosomatids protozoans RNAi was absent in both *Leishmania major* and *Trypanosoma cruzi*. The genome of *L. braziliensis*, a member of the early diverging *Leishmania* subgenus *Viannia*, retained key genes required for RNAi such as an *Argonaute*. We demonstrated that in fact *L. braziliensis* shows strong RNAi activity with reporter and endogenous genes affecting flagellar function. These data suggest that RNAi may be productively applied for functional genomic studies in *L. braziliensis*. We mapped the evolutionary point at which RNAi was lost in lineage leading to *Leishmania* and *Crithidia*, and establish that RNAi must have been lost at least twice in the trypanosomatids, once on the lineage leading to *T. cruzi* and independently following the divergence of the *Viannia* subgenus from other *Leishmania* species. Lastly, we discuss hypotheses concerning the forces leading to the loss of RNAi in *Leishmania* evolution, including viral invasion, increased genome plasticity, and altered virulence.

## Introduction

In metazoans, RNAi interference and related pathways play many key roles including regulation of mRNA levels and translation, chromatin silencing, programmed DNA rearrangements, genome surveillance, and defense against invading viruses. The phylogenetic distribution of key genes required for RNA interference such as *Argonaute* and *Dicer* suggests that this pathway may have been present in the common eukaryote ancestor [1]. However the situation for eukaryotic microbes is complex: some have active RNAi pathways, others lack RNAi genes and activity, and demonstration of RNAi has proven elusive in some species bearing reasonable homologs of canonical genes such as *Argonaute* [2]–[7].

The trypanosomatid protozoa comprise three major lineages, broadly grouped as the African trypanosomes (*Trypanosoma brucei*), South American trypanosomes (*T. cruzi*) and a lineage encompassing a number of genera associated with insects or plants, ultimately leading to the mammalian parasite *Leishmania* [8]. Functional and genome sequencing data have shown that species within the African trypanosome lineage such as *T. brucei* contain an active RNAi pathway and genes, including an *Argonaute* “slicer” (*AGO1*; [2]) and two Dicers (*DCL1* and *DCL2*; [9], [10]). In contrast, *T. cruzi*, *L. major* and *L. donovani* lack these activities and associated genes [11]–[14]. However the genome of *L. braziliensis* (subgenus *Viannia*) contains orthologs of *T. brucei AGO1*, *DCL1* and *DCL2* [15], suggesting this group might retain a functional RNAi pathway. Given the uncertainties of extrapolating from RNAi genes to functions noted in other eukaryotic microbes [2]–[4], we sought to establish whether the RNAi machinery functions in *L. braziliensis*, and explored its utility as a genetic tool. Furthermore, we made evolutionary comparisons to map when the RNAi pathway was lost, and we discuss potential selective forces impacting on the parasite that may have contributed to the demise of RNAi during *Leishmania* evolution.

## Results

### siRNA formation in *L. braziliensis*

Dicer is required to process long dsRNA to small interfering RNAs (siRNAs), which in trypanosomes are 24–26 nt long [16]. A convenient marker of RNAi activity is siRNA formation from endogeneous retroelements [17], and Northern blot analysis of *L. braziliensis* RNAs revealed the presence of small RNAs of the expected sizes arising from the retroelement *SLACS*, similar to *T. brucei* siRNAs (Fig. S1; [16]).

We then developed a green fluorescent protein (GFP)-based RNAi reporter assay for siRNA formation, as well as target mRNA and protein levels. Initially we experienced unexpected difficulty in *L. braziliensis* transfection, when using episomal constructs previously developed in one of our labs that function effectively in many *Leishmania* species, and in many laboratories [18]. The basis for this effect is not definitively known, as addressed in the discussion, but we suspect it is due to the tendency of episomal vectors to be transcribed from both strands, which in an RNAi-proficient species would strongly inhibit episomal gene expression [11], [13]. Thus in all studies reported here, transfection was accomplished following integration of DNA constructs into the ribosomal small subunit RNA (*SSU*) locus, using the appropriately digested DNA from pIR1SAT-based vectors, or derivatives thereof [19]. In trypanosomatids, processing of polycistronic RNA precursors by 5′ *trans*-splicing and 3′ polyadenylation produces capped mRNAs that can direct protein synthesis [20].

**Figure 1:**
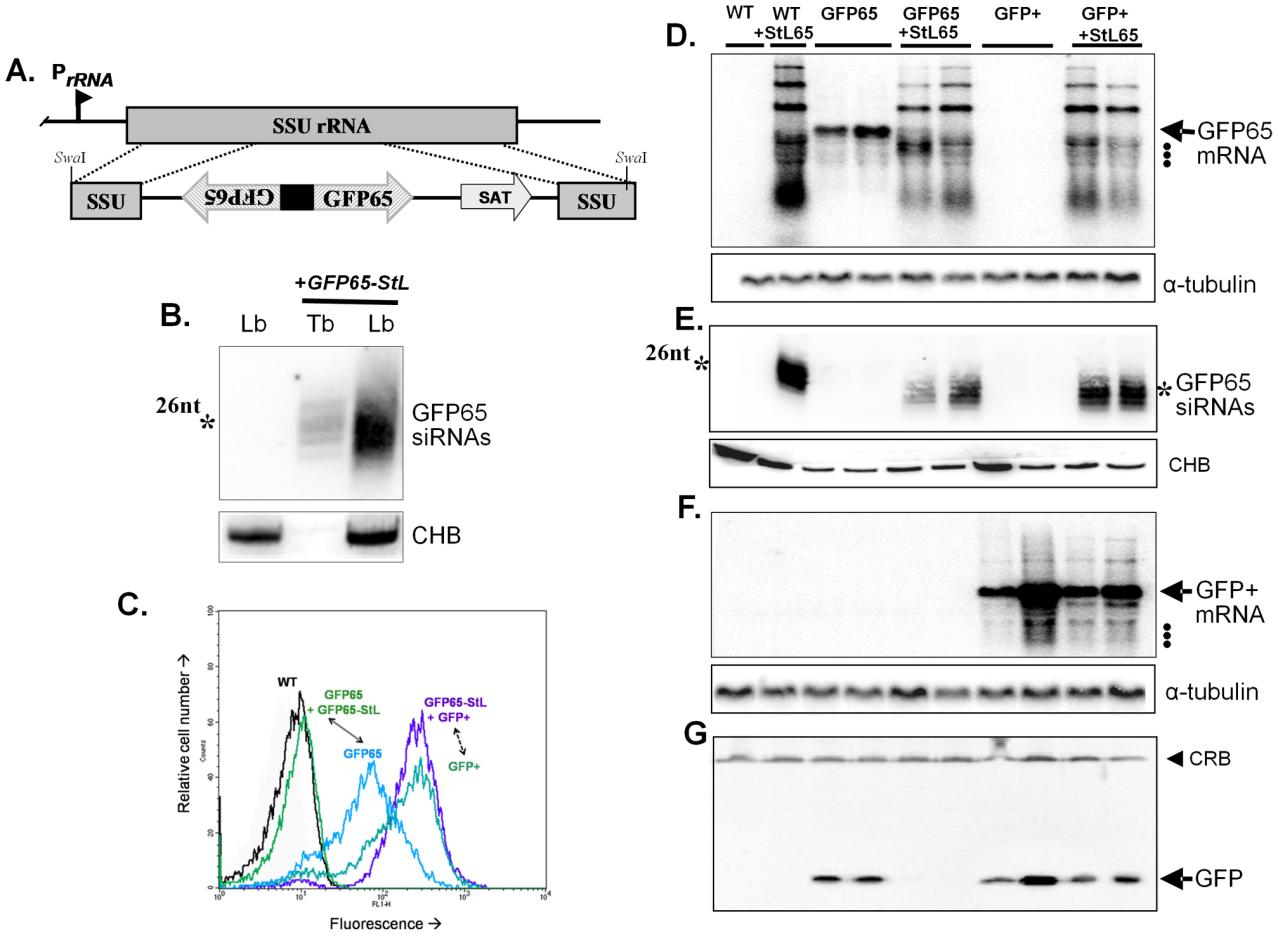
Tests of RNAi pathway activity in *L. braziliensis* using GFP reporters. **Panel A**. Schematic map of the *SSU* rRNA locus in *Leishmania* and an example of targeting using the *Swa*I GFP65-StL fragment derived from the *GFP65-StL* construct pIR1SAT-GFP65-StL. Regions are the SSU rRNA (gray box), *GFP65* ORF (striped arrow), nourseothricin resistance gene ORF (SAT), stem-loop stuffer fragment (black box), and the rRNA promoter (P_rRNA_). **Panel B**. siRNA analysis of WT *L. braziliensis* M2903 and GFP65-StL transfectants of *T. brucei* [63] and *L. braziliensis* M2903 *SSU:GFP65-StL*, hybridized with a radiolabeled GFP65 probe. The star marks the mobility of a 26 nt standard; CHB is a cross hybridizing band that serves as a loading control. **Panel C**. GFP flow cytometry of *L. braziliensis* M2903 transfectants expressing either the AT-rich GFP65* or the GC-rich *GFP*+ reporters, alone or in combination with a *GFP65-StL*. Profiles are labeled and color-coded as follows: Black, WT M2903; green, *GFP65+GFP65-StL* (SSU:GFP65-StL + SSU:GFP65*, clone 8); blue, *GFP65* (SSU:GFP65*, clone 10); blue-green, GFP+(*SSU:GFP+*, clone 38); and purple, *GFP65-StL + GFP+ *(*SSU:GFP++SSU:GFP65-St*L, clone 60). **Panel D**. Northern blot analysis of *L. braziliensis* M2903 derived lines; WT, *SSU:GFP65-StL*, *SSU:GFP65*, *SSU:GFP65 + SSU:GFP65-St*L, *SSU:GFP+*, and *SSU:GFP++SSU:GFP65-StL*. The hybridization probe was radiolabeled *GFP65*. Hybridization with a α-tubulin probe was used as a loading control and the migration of rRNAs (1.5, 1.8 and 2.2×103 nt; see GenBank AC005806) are indicated by dots. **Panel E**. siRNA analysis of lines described in panel C, probed with radiolabeled GFP65. The star marks the mobility of a 26 nt standard and CHB is a cross hybridizing band that serves as a loading control. **Panel F**. Northern blot of analysis of lines described in Panel C, hybridized with the GC-rich GFP+ probe. Hybridization with a α-tubulin probe was used as a loading control and the migration of rRNAs are indicated by dots. **Panel G**. Western blot of lines described in panel C probed with anti-GFP antisera. The filled arrowhead indicates a cross-reactive band (CRB) that serves as a loading control.

First we generated a GFP ‘stem-loop’ (long hairpin) construct, containing two copies of an AT-rich GFP reporter (GFP65) in an inverted orientation separated by a short loop (Fig. 1A). This GFP stem-loop construct (*GFP65-StL*) was flanked by *Leishmania* sequences required for efficient 5′ and 3′ end mRNA formation, and was expressed following integration into the parasite small subunit ribosomal RNA locus (*SSU* rRNA; Fig. 1A) in *L. braziliensis* strain M2903.

Northern blot analysis with a *GFP65* probe showed that expression of *GFP65-StL* gave rise to a variety of products (Fig. 1D, lane 2). The largest of these likely correspond to unprocessed transcripts, while the smaller ones likely correspond to degradation products, which could occur irrespective of whether RNAi pathways are active. Importantly, abundant levels of 24–26 nt siRNAs were seen (Figs. 1B and 1E). In contrast, similarly small RNAs were not detected with probes to the *SAT* drug resistance marker, which is not found in an inverted repeat (data not shown). These data suggested that *L. braziliensis* expresses a robust Dicer-like activity.

### Demonstration of RNAi activity

We used two GFP reporters, one encoded by the AT-rich ORF (*GFP65*) used in the GFP65-StL construct above, and the second by a GC-rich ORF (*GFP+*). These genes differ in most 3^rd^ codon positions, but their protein products only differ by a single amino acid. Alignment of these genes showed that the longest tracts of identical nucleotides were less than 14 nt (Fig. S2). *GFP65* or *GFP+* was then expressed separately following integration into the *SSU* rRNA locus, in wild-type (WT) *L. braziliensis* or the *GFP65-StL* transfectant that produces GFP65 siRNAs.

As expected, expression of *GFP65* or *GFP+* led to high levels of GFP mRNA and protein in WT lines, as did expression of *GFP+* within the *GFP65-StL* transfectant (Fig. 1D, F, G). In contrast, clonal lines arising from introduction of *GFP65* into the *GFPST-StL* transfectant showed only trace amounts of *GFP65* mRNA (Fig. 1D), and the level of GFP protein was below the limit of detection by western blotting (<1% in these studies; Fig. 1G) or flow cytometry (Fig. 1C). These data established that GFP65-derived dsRNA mediated selective ablation of the AT-rich GFP65 but not the GC-rich GFP+.

Similar studies were carried out with a luciferase (*LUC*) reporter, expressed alone or in combination with a *LUC* stem-loop construct, revealing strongly-reduced LUC expression (90–300 fold; Fig. S3, and other studies below).

### RNAi activity against endogenous *L. braziliensis* genes

**Figure 2:**
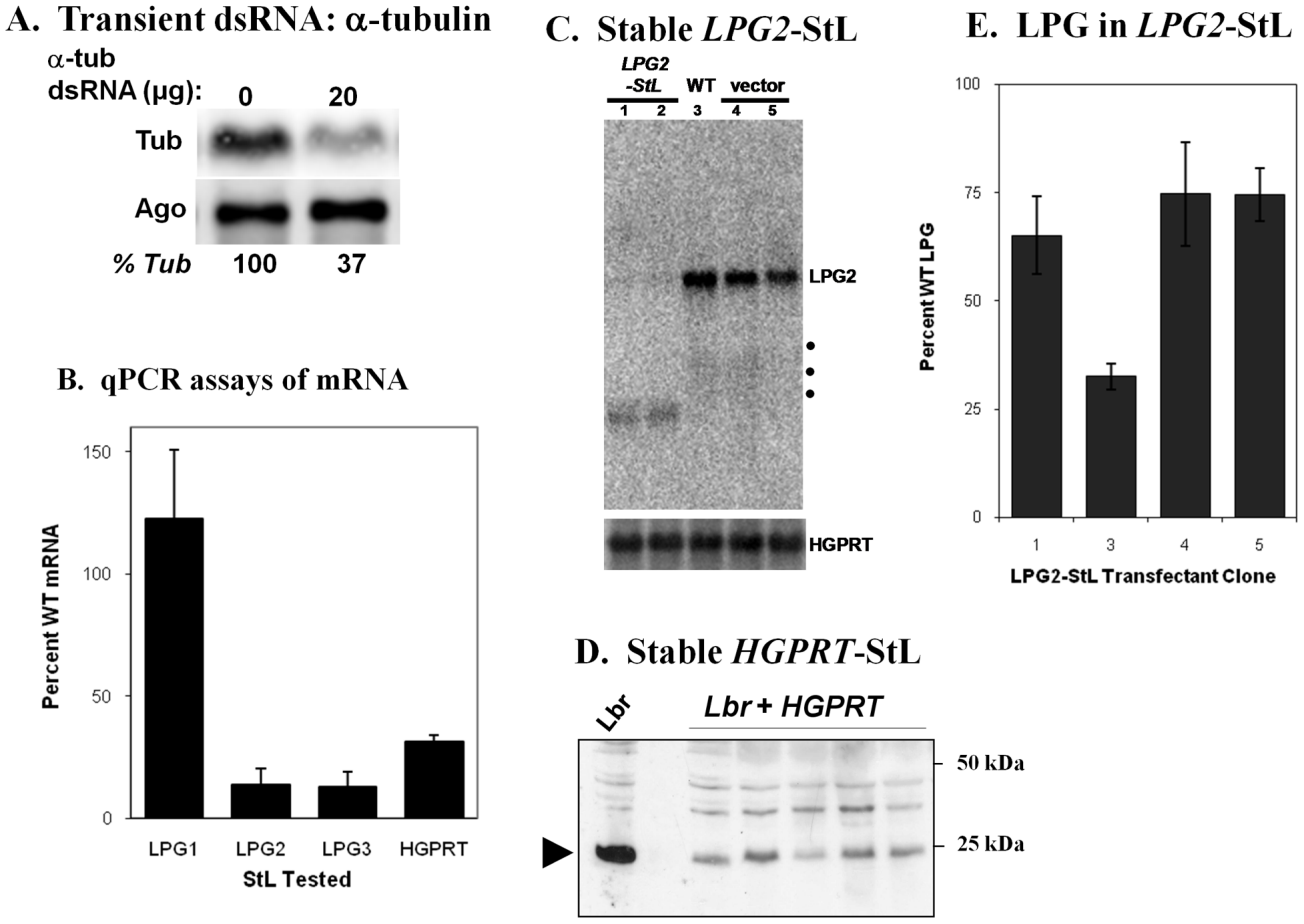
RNAi of endogeneous *Leishmania braziliensis* genes: effects on mRNA, protein or LPG expression. **Panel A**. Northern blot analysis of *L. braziliensis* transiently transfected with α-tubulin dsRNA. Cells were electroporated with 20 µg dsRNA from the *T. brucei* paraflagellar rod protein gene (lane 0) or with 20 µg dsRNA derived from *L. braziliensis* α-tubulin (lane 20). **Panel B**. mRNA levels of *LPG1-StL*, *LPG2-StL*, *LPG3-StL* or *HGPRT-StL L. braziliensis* M2903 stable transfectants, as determined by quantitative RT-PCR, relative to WT and/or control transfectants. The average and standard deviation from 4–6 transfectants for each construct are shown. **Panel C**. Northern blot analysis of *LPG2-StL* transfectants. Total RNAs were hybridized with a radiolabeled *L. braziliensis LPG2* probe, located outside of the *LPG2* ‘stem’. Lane 1, *LPG2-StL*-F; lane 2, *LPG2.-StL*-R transfectant; lane 3, WT M2903; lanes 4 and 5, empty vectors (*StL-*F and *StL-*R, respectively). **Panel D**. Western blot of HGPRT protein; Lbr, M2903; Lbr+*HGPRT-StL*, independent Lbr *SSU:HGPRT-StL* transfectants. **Panel E**. LPG in *LPG2-StL* transfectants. LPG was isolated from WT and independent *SSU:LPG2-StL* transfectants and quantitated, and expressed as percent WT levels.

We then tested the activity of the RNAi pathway on several endogenous genes. In transient transfections performed using several protocols and dsRNAs synthesized *in vitro* against the *L. braziliensis* α-tubulin, Northern blot analysis showed at best a 63% decrease in α -tubulin mRNA (Fig. 2A). This contrasts with *T. brucei* where such protocols readily yield >95% reduction in tubulin mRNA expression [21]. This perhaps reflects the lower efficacy of transient transfection attained thus far in *Leishmania* [11].

Since inducible expression systems were unavailable, we focused on stably expressed ‘stem-loop’ constructs targeting a panel of nonessential genes in *L. braziliensis*, including ones mediating synthesis of the abundant glycoconjugate lipophosphoglycan (*LPG1*, *LPG2*, *LPG3*; [22]), hypoxanthine-guanine phosphoribosyltransferase (*HGPRT*), or the genes *PFR1* and *PFR2*, which encode major components of the paraflagellar rod, a component of the trypanosomatid flagellum required for motility [23]. These *StL*-transfectants showed a variable decrease in mRNA levels when estimated by qPCR, ranging from no effect (*LPG1*) to more than 10-fold reduction (*LPG2*, *LPG3*; Fig. 2B). However, Northern blot analysis showed a nearly complete absence of *LPG2* mRNA (Fig. 2C), suggesting that the qPCR values are likely underestimates, possibly due to the presence of RNA degradation intermediates able to act as templates (these are evident in Fig. 2C). Despite the reductions in mRNA levels, LPG levels were at best only 3-fold lower in the *LPG2-StL* or *LPG3-StL* transfectants, with considerable clonal variability (Fig. 2E; data for *LPG3-StL* not shown). This suggests that *L. braziliensis* requires only low levels of LPG biosynthetic proteins, similar to the relatively small effects of RNAi on trypanosome glycoconjugate biosynthetic genes [24]. Both *HGPRT* mRNA and protein levels showed 3–4 fold decreases in *HGPRT-StL* transfectants (Fig. 2B, D).

**Figure 3:**
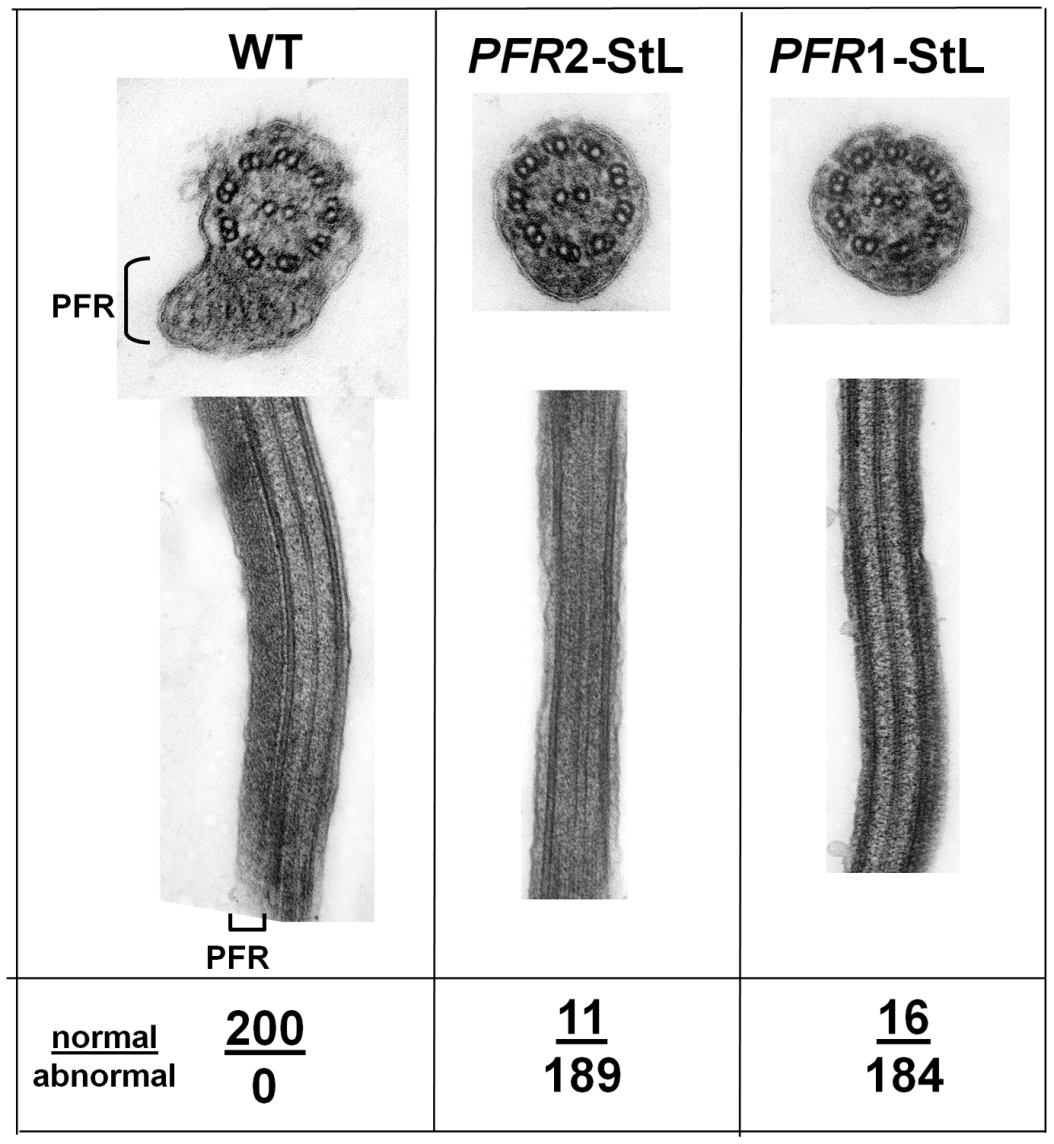
Ablation of paraflagellar rod synthesis following RNAi of *PFR1* or *PFR2*. *L. braziliensis* M2903 was transfected with constructs expressing *PFR1-StL* or *PFR2-StL* via integration into the *SSU* rRNA locus yielding clonal lines with typical transfection frequencies. Several of these, along with WT, were fixed, stained and subjected to transmission electron microscopy as described in the methods. The location of the paraflagellar rod adjacent to the flagellar axonemes is shown (PFR); its presence or absence was scored in 200 cells as indicated below the figure.

One of the earliest reports of stable phenotypic modulation by RNAi in trypanosomes involved down regulation of a paraflagellar rod protein [25], [26]. The paraflagellar rod is a complex assembly of proteins required for motility, which in trypanosomatids includes two major proteins, termed PFR1 and PFR2 in *Leishmania* [23], [27], [28]. Introduction of *PFR1-StL* or *PFR2-StL* constructs into *L. braziliensis* yielded viable transfectants that grew normally, but lacked the paraflagellar rod, as visualized in longitudinal or transverse EM sections, and exhibited motility defects (Fig. 3). These phenotypes closely resemble those seen in *L. mexicana PFR1* and *PFR2* gene deletion mutants [23].

Multiple attempts to introduce ‘stem-loop’ α- or β-tubulin constructs were unsuccessful, as anticipated for essential genes (not shown). Collectively, the strength of the RNAi effect for these phenotypic reporters suggests that RNAi may function sufficiently well to assess the functions of many genes in *L. braziliensis*.

### RNAi of *AGO1* establishes its role in the RNAi pathway in *L. braziliensis*

**Figure 4:**
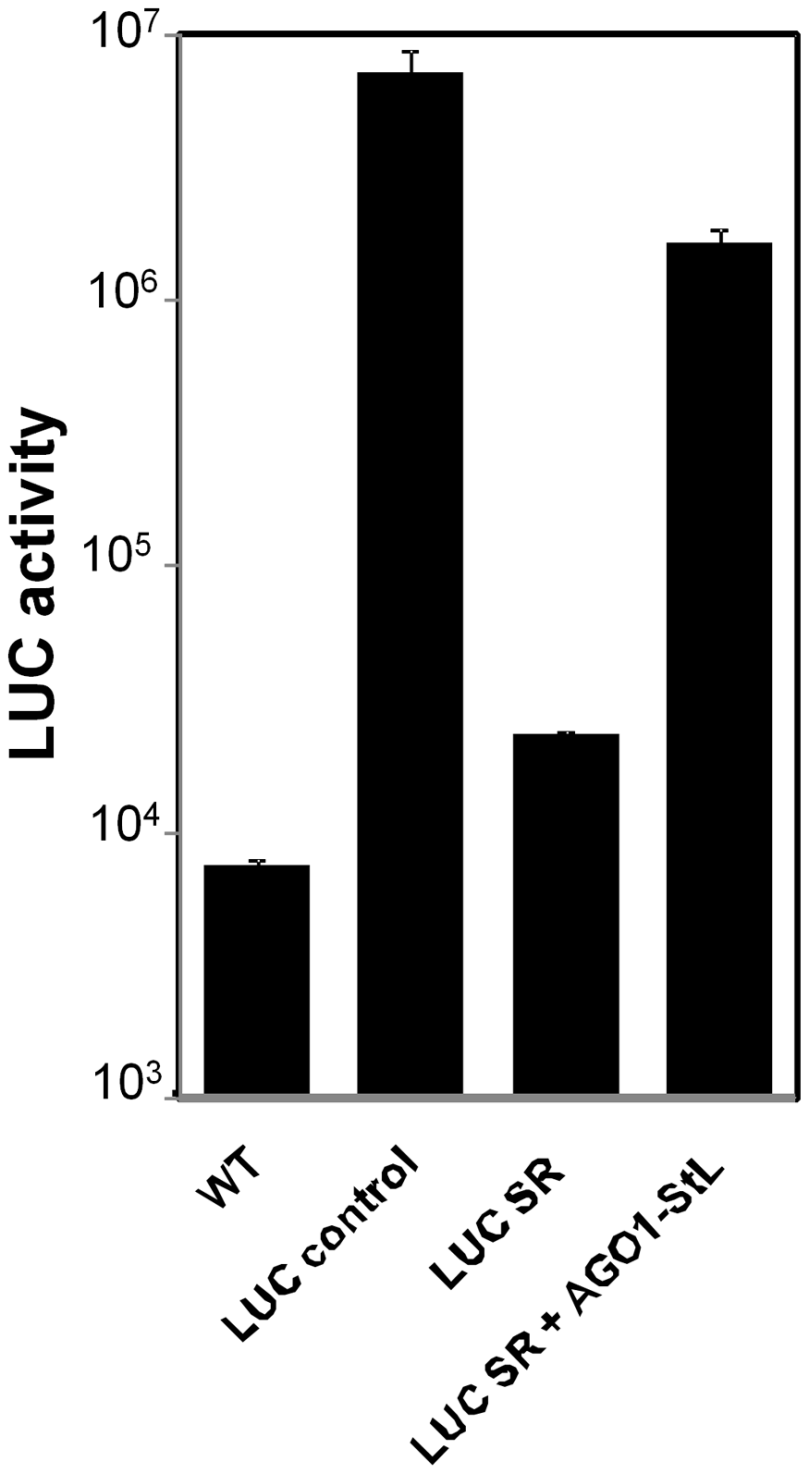
RNAi of *AGO1*. LUC assays of *L. braziliensis* M2903 lines bearing the indicated constructs. WT, *L. braziliensi*s M2903; LUC control, *SSU::IR2HYG-LUC(b)*; LUC SR, *SSU:IR2SAT-LUC-StL(a)-LUC(b)*; LUC SR + AGO1 StL, *SSU:IR2HYG-LUC-StL(a)-LUC-(b)* + *SSU:IR1SAT-AGO1-StL(b)*. Standard deviations are shown; measurements were made in triplicate of the control lines, while the LUC SR+ AGO1 StL represents the average of 12 independent clones, each measured in duplicate.

In other organisms RNAi is mediated by the combined activity of a number of proteins, ultimately converging on the endonucleolytic ‘slicer’ activity of the Argonaute protein, which is encoded by the single *AGO1* gene in trypanosomes and *L. braziliensis* [15], [17]. To establish a critical role for *L. braziliensis AGO1* in RNAi, we employed the seemingly counterintuitive approach of ‘RNAi of RNAi genes’, where introduction of dsRNAs targeting RNAi pathway genes inhibits RNAi activity, albeit not to the same level seen in null RNAi pathway gene knockouts [17], [29]–[31]. To facilitate comparisons of the efficacy of RNAi, we developed a single RNAi ‘self reporter’ construct which simultaneously expressed two mRNAs, one encoding a luciferase ORF (*LUC*) and a second encoding a luciferase ORF stem-loop (*LUC*-*StL*). This minimized experimental variability and the number of transfections required, allowing the assessment of RNAi efficacy by the introduction of a single construct. When introduced into WT *L. braziliensis*, the ‘*LUC* RNAi self reporter’ (*LUC*-SR) showed low levels of luciferase activity, about 4-fold over background and comparable to that obtained with lines expressing *LUC* and *LUC-StL* independently after successive transfections (Fig. 4). In contrast, introduction of the *LUC* reporter alone resulted in activities nearly 1000-fold over background (Fig. 4).

We then introduced a construct expressing an *AGO1* stem-loop (*AGO1-StL*) into the *LUC* RNAi reporter line (*LUC*-SR). These transfectants showed an average of 100-fold increased luciferase expression relative to *LUC*SR transfectants, signifying a considerable reduction in the efficiency of RNAi (Fig. 4). As expected from studies in other organisms cited above, inhibition of RNAi activity was partial, as these values were still about 10-fold less than seen in WT cells transfected with the *LUC* reporter construct alone (Fig. 4). These data thus implicate *AGO1* as an essential component of the RNAi pathway of *L. braziliensis*.

### Mapping of the point in *Leishmania* evolution at which RNAi activity and RNAi pathway genes were lost

**Figure 5:**
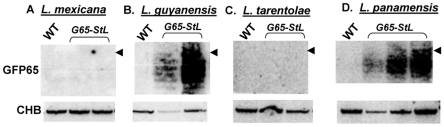
GFP siRNAs in *Leishmania* species. The indicated species were electroporated with the targeting fragment from pIR1SAT-GFP(65)-StL, yielding *SSU:SAT-GFP(65)-StL* transfectants. These were confirmed by PCR tests for the marker and presence of the inverted GFP65 repeats, and RNA was isolated and subjected to Northern blotting for siRNAs using a GFP65 probe. CHB indicates a cross hybridizing band that serves as a loading control, and the arrow head indicates the position of a 26 nt DNA marker. Panel A and B samples were run on one gel, Panel C and D samples on another one.

We explored the prevalence of RNAi pathways in other Trypanosomatid species by comparative genomics. PCR assays detected *AGO1* and/or *DCL1* genes in all isolates of the *Leishmania* subgenus *Viannia* tested (*L. braziliensis*, *L. guyanensis*, *L. panamensis*) but not in *Leishmania* (*Sauroleishmania*) *tarentolae*, *L. mexicana*, *L. major* or *L. donovani* (data not shown). Partial genome sequencing of a close non-parasitic ‘outgroup’, *Crithidia fasciculata* revealed *AGO1*, *DCL1* and *DCL2*. To confirm the presence or absence of a functional RNAi pathway, we expressed the *GFP65-StL* RNA in *L. tarentolae*, *L. mexicana*, *L. panamensis*, *L. guyanensis* and *Crithidia fasciculata*, and monitored siRNA formation by Northern blotting. Consistent with the observed distribution of RNAi pathway genes, GFP siRNAs were made only in *Crithidia*, *L. guyanensis* and *L. panamensis* (Fig. 5, S4). Transfection with the GFP reporters showed strong reductions in GFP expression in *L. panamensis*, comparable to that seen with *L. major* in Fig. 1 (data not shown), and we show in a later section that RNAi is active in *L. guyanensis* using a luciferase reporter The level of GFP expression in *Crithidia* with the *Leishmania* vectors used was too low to utilize for quantification of the strength of RNAi by flow cytometry (data not shown).

**Figure 6:**
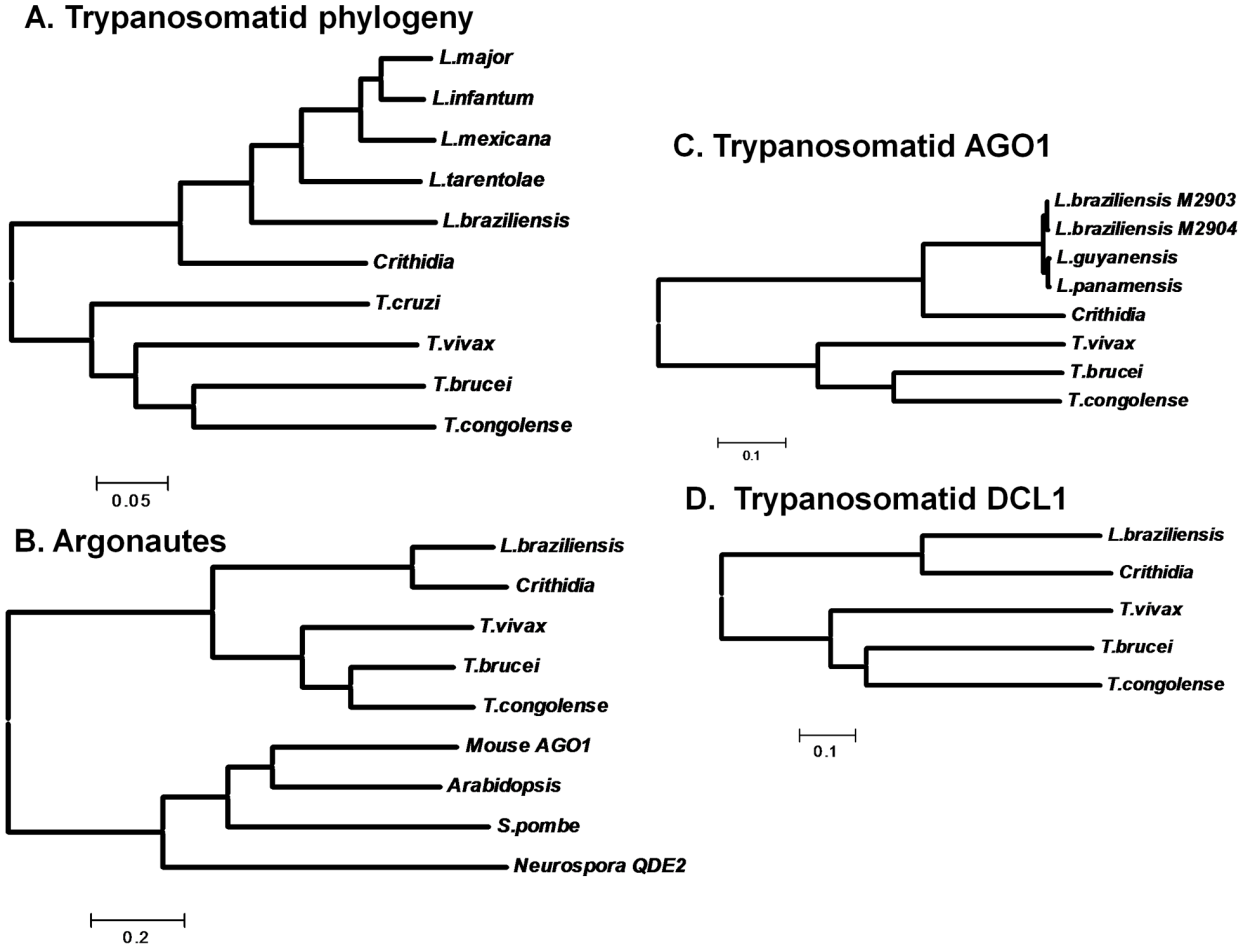
Evolutionary tree of trypanosomatid housekeeping genes, AGO1s and Dicers. **Panel A**. Protein-based phylogeny of trypanosomatid species considered in this work. We identified the predicted protein sequences for PTR1, (pteridine reductase 1), GSH1 (γ-glutamylcysteine synthetase) and APRT (adenine phosphoribosyl transferase) in public databases (www.genedb.org) or preliminary genome sequence assemblies from *Crithidia fasciculata*. For each species the three protein sequences were concatenated, aligned using the ClustalW algorithm, and a neighbor joining tree was generated using the MEGA4 software [64]. The scale corresponds to inferred number of amino acid substitutions. The tree shown agrees well with consensus evolutionary trees presented elsewhere [52]. **Panel B**. Argonautes. A molecular tree was created as described in the legend to Panel A using representative metazoan Argonaute sequences as well as *T. brucei* AGO1, *L. braziliensis* AGO1, *Crithidia fasciculata* AGO1 (this work), and predicted AGO1s for *T. congolense* and *T. vivax* (www.genedb.org). **Panel C**. Trypanosomatid Argonautes. A molecular tree was generated as described in panel B, including only the eight trypanosomatid AGO1s. **Panel D**. Trypanosomatid *DCL1*s. A molecular tree was generated as described in panel B, including only the five sequenced trypanosomatid DCL1s.

**Figure 7:**
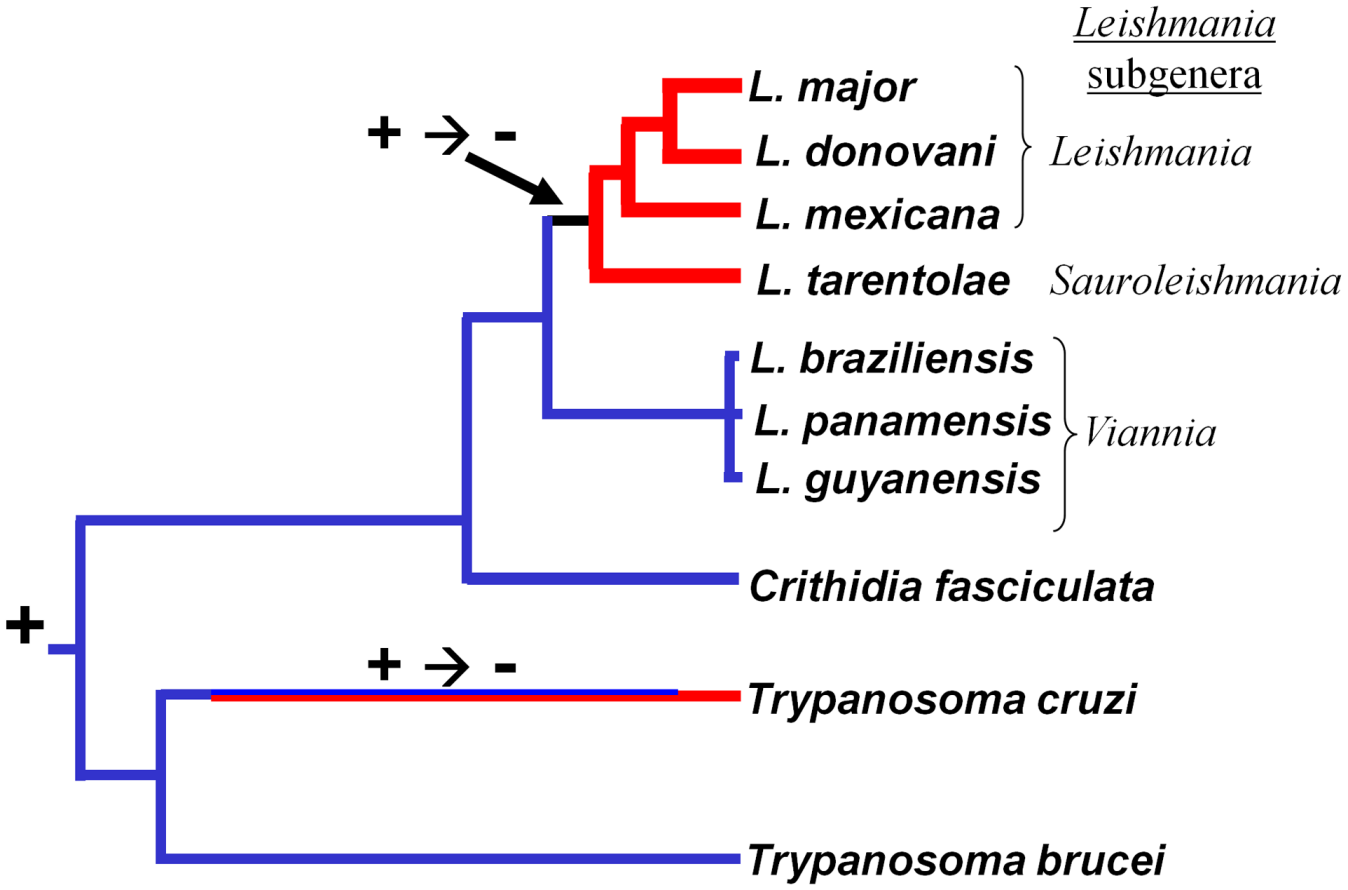
Retention and loss of RNAi machinery and activity during trypanosomatid evolution. A consensus evolutionary tree is shown; the scale corresponds to the degree of evolutionary divergence amongst these organisms (Fig. 6). Lineages lacking RNAi activity and/or genes are indicated at the termini, with RNAi-deficient lineages colored red and RNAi-proficient lineages colored blue. Bicoloring along the *T. cruzi* lineage signifies that the point at which RNAi was lost is unknown. The + or − symbols indicate the presumptive presence and/or loss of RNAi during evolution.

Association of these findings with the trypanosomatid evolutionary tree (Fig. 6A) through evolutionary parsimony identified a single point when the RNAi pathway was lost during evolution, located after the divergence of members of the subgenus *Viannia* from the remaining species complexes (Fig. 7). Importantly, this corresponds precisely to the point when RNAi genes were lost in evolution, as deduced by comparative genomics and evolutionary parsimony. Inspection of the sequenced *Leishmania* genomes shows that all RNAi-deficient *Leishmania* now contain only remnant, highly degenerate pseudogenes (*AGO1*) or have undergone gene deletion (as revealed by ‘synteny gaps’ for *DCL1* and *DCL2*) for known trypanosomatid RNAi genes. Since species retaining only a partial set of intact RNAi genes have not been reported, from these data we cannot identify which essential RNAi pathway gene was lost first at this distant point in *Leishmania* evolution. Presumably, once a gene critical for RNAi activity was inactivated, the remaining genes of the pathway become superfluous and fall prey to evolutionary drift, as seen in many other metabolic pathways during evolution.

RNAi pathways were probably present in the common eukaryote ancestor [1], and the evolutionary relationships of the available trypanosomatid RNAi pathway proteins closely resemble those of housekeeping protein-based phylogenies (shown for AGO1 and DCL1 in Fig. 6 B–D). While the *L. braziliensis AGO1* gene is not syntenic with that of *T. brucei* [15], [32] the congruency of the RNAi gene and ‘housekeeping’ gene phylogenies renders the possibility of lateral gene transfer and/or independent acquisitions unlikely. Thus, RNAi most likely was lost twice independently in trypanosomatids, once in the lineage leading to *T. cruzi*, and a second time in the lineage leading to *Leishmania*, subsequent to the divergence of most *Leishmania* groups from the non-parasitic species *Crithidia fasciculata* and the *Leishmania* subgenus *Viannia* (Fig. 7).

### RNAi activity in virus+ vs. virus-free *Leishmania*

We and others have speculated that one of the forces contributing to the loss of RNAi in eukaryotic microbes may be invasion or loss of RNA viruses [13], [33]. Significantly, dsRNA viruses termed LRVs are found in many (but not all) strains and/or species from the *Leishmania* subgenus *Viannia*, including *L. braziliensis* [34], [35]. We reasoned that studies of the efficacy of RNAi in extant *Leishmania* bearing or lack LRVs could provide some insight into their potential roles in evolution.

**Figure 8:**
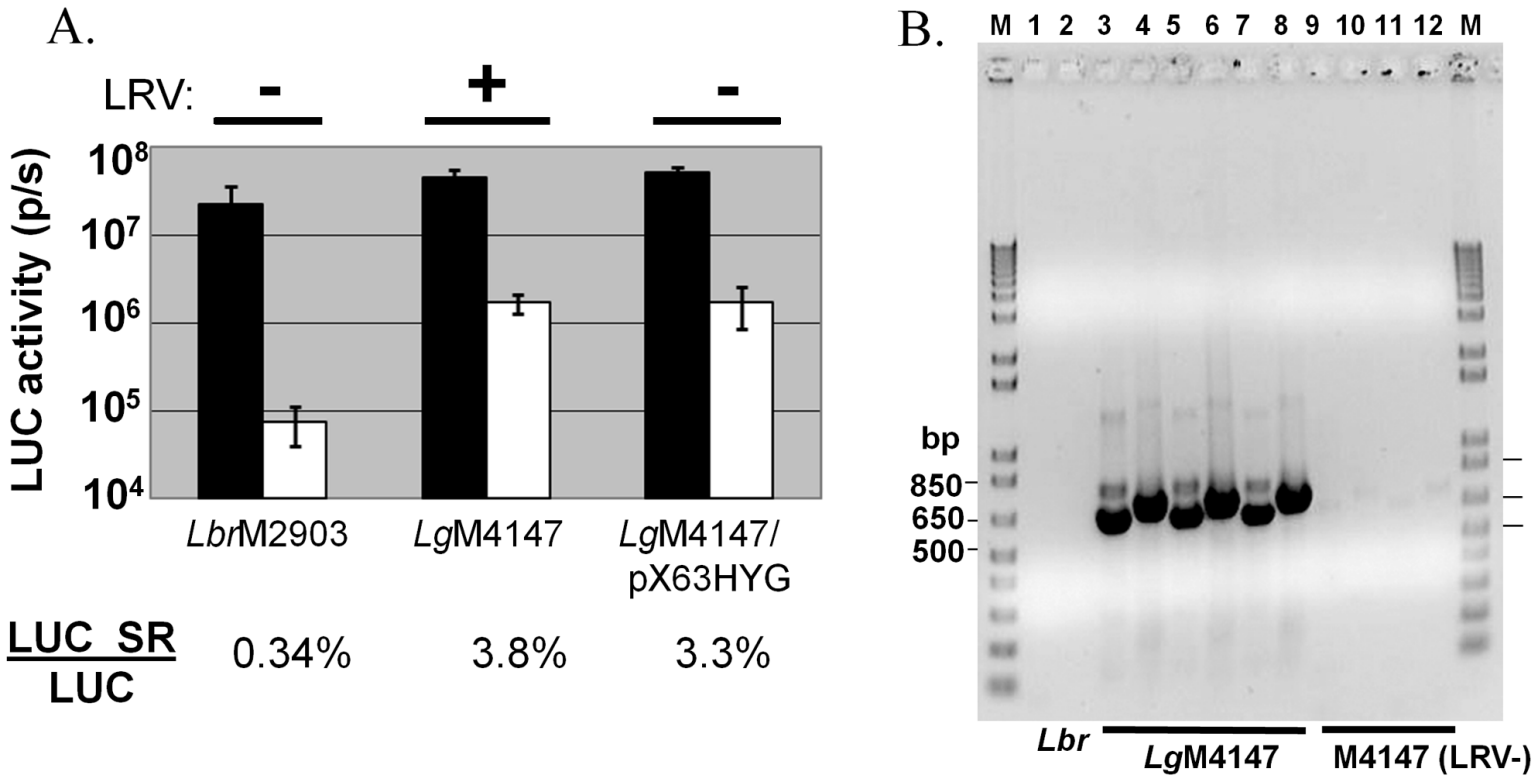
Efficiency of RNAi is reduced in *L. guyanensis* M4147 independent of LRV status. **Panel A**. *LUC* RNAi reporter assays. pIR2SAT constructs expressing *LUC* alone (black boxes) or the *LUC* RNAi self reporter (LUC SR; white boxes) were introduced separately into *L. braziliensis M2903*, *L. guyanensis* M4147 (LRV1-4 virus-containing), *or L. guyanensis* M4147/pX63HYG (virus-free). *SSU*-integrated clonal lines were obtained and assayed for luciferase activity (n = 4 for M2903; n = 6 for *L. guyanensis*; the average and standard deviations are shown). The ratio of luciferase activities between the LUC SR and LUC expressing clones of each of the three lines are shown below the graph. **Panel B**. PCR confirmation of LRV1-4 virus status in parental and transfectant *L. guyanensis* M4147. PCR primers were LRV1-4 set 1 (lanes 3,5,7,9,11) or set 2 (lanes 2,4,6,8,10,12) (Table S1). RT-PCR reactions were performed with RNAs isolated from *L. braziliensis* M2903 (virus-free control; lanes 1,2), M4147 (obtained from two sources; lanes 3,4 and 5,6), M4147+LUC SR (lanes 7,8), M4147/pX63HYG (lanes 9,10), or M4147/pX63HYG + LUC SR (lanes 11,12). M, molecular size marker.

Using specific PCR primers for LRVs we showed that the *L. braziliensis* strain M2903 used here lacked LRVs, consistent with previous reports [36], [37]. Unfortunately methods for the introduction and/or cure of LRV from *Leishmania* are not well developed, precluding tests of isogenic *L. braziliensi*s engineered to harbor the LRV virus. Similarly, just one isogenic virus-free derivative of an LRV-containing *Leishmania* has been described; *L. guyanensis* is closely related to *L. braziliensis* (Fig. 7), and a virus-free derivative arose fortuitously in the course of other studies [38]. The efficiency of RNAi in these lines was evaluated by introduction of the luciferase RNAi reporter (*LUC*-SR) described earlier, relative to transfectants expressing only *LUC*. Multiple clonal lines were obtained, and LUC expression was measured in six randomly selected lines (Fig. 8A). Importantly, the level of luciferase expression seen in the lines expressing only *LU*C were comparable between the closely related *Viannia* species M2903 *L. braziliensis* and M4147 *L. guyanensis* (Fig. 8A). All lines and transfectants were shown to retain or lack the LRV1-4 by RT-PCR tests as expected (Fig. 8B).

While the RNAi pathway was active in the LRV+ *L. guyanensis* M4147, its efficiency was only about 30-fold (3.8% LUC-SR vs. LUC), compared to the 300-fold reduction seen in the virus free *L. braziliensis* M2903 (0.34% LUC-SR/LUC; Fig. 8A). The WT LRV+ *Lg*M4147 strain also showed reduced efficiency of RNAi relative to M2903, in studies using successively transfected GFP reporter and GFP-StL constructs (data not shown). Significantly, the LRV-free line *Lg*M4147/pX63HYG showed a similar 30-fold efficiency of RNAi in these studies (3.3% LUC SR/LUC). These data suggest that the reduced RNAi efficiency seen in *L. guyanensis* M4147 does not require the continued presence of the virus.

## Discussion

### *L. braziliensis* has a strongly active RNAi pathway able to reduce target gene expression

Our studies have established that *L. braziliensis* possesses a functional RNAi pathway, which enables the down-regulation of a variety of reporter and endogenous genes when assayed at the mRNA or protein levels. RNAi of *AGO1* was used to confirm a requirement for the sole argonaute gene *AGO1* in this process. As seen in many organisms, strong reductions in mRNA expression were seen, often accompanied by phenotypic changes, albeit of variable strength. As anticipated, it was not possible to introduce stem-loop constructs for essential genes such as α- or β-tubulins. Studies of such genes will require the development of inducible expression systems in *Leishmania*, which while promising have not yet reached the point of utility attained in trypanosomes.

Strong phenotypes were produced by the knockdown of two genes implicated in flagellar motility and paraflagellar rod synthesis (*PFR1* and *PFR2*), closely approximating the phenotypes seen in gene deletion mutants in *L. mexicana* [23]. In contrast, at best only weak phenotypes were produced by knockdowns of three LPG biosynthetic genes, in keeping with findings in trypanosomes where it has proven difficult to down-regulate expression of genes implicated in glycoconjugate synthesis far enough to attain phenotypic effects. Overall, the results to date suggest that the range in efficacy of RNAi knockdowns, as judged by various phenotypic criteria, is comparable to that seen in trypanosomes and other organisms, and thus is likely to be similarly useful in the systematic analysis of *Leishmania* gene function in the future.

### Factors potentially impacting on the evolutionary loss of RNAi in *Leishmania*

Given the importance of RNAi pathways in many fundamental aspects of eukaryotic biology, it is unsurprising that it has been lost relatively few times during evolution. While the critical roles of RNAi in metazoan gene regulation would likely select strongly against such attenuation, eukaryotic microbes lacking RNAi have arisen sporadically [1], [2]. This in turn raises the question of under what circumstances RNAi might occur. We consider three working hypotheses for selective pressures that may act independently or in concert to drive this loss in *Leishmania*.

### Viruses

We proposed previously that viral pressure could act as a selective force for the loss of RNAi in *Leishmania* evolution [11], [13]. In one scenario, invasion by LRVs at some point in *Leishmania* evolution could lead to an attenuation of the RNAi response, as many RNA viruses are prone to attack by cellular RNAi pathways [39]. Attenuation could be achieved through down regulation of the RNAi pathway by the host cell, or through viral genes targeting key RNAi pathway activities. While some RNA viruses encode inhibitors of RNAi, no studies have been undertaken as yet for *Leishmania* LRVs. The challenge for this model is to explain what forces would prompt cells to favor RNA virus retention over disruptions arising from perturbation or loss of the RNAi pathway. Interestingly, LRV infection has been proposed to be advantageous to *Leishmania*, possibly by modulating host immune responses in a way beneficial to parasite survival [40], [41]. In support of this hypothesis, recently we have obtained preliminary in support of the proposal that LRV-containing *L. guyanensis* show increased survival and pathogenicity (L-FL, KO, S. Hickerson and SMB, unpublished data; N. Fasel, personal communication). Selection for the presence of LRV able to promote parasite survival could thus provide a selective force promoting down-regulation of RNAi activity targeting RNA viruses.

While one cannot perform experimental tests in the ancestral *Leishmania*, one prediction is that in extant species or strains now harboring *Leishmania* LRVs, attenuation of the RNAi response may occur. Here we compared the efficacy of RNAi seen in the virus-free *L. braziliensis* M2903 used in the majority of our studies with a closely related species *L. guyanensis* that bears the cytosolic dsRNA virus LRV1-4 [35], [36] (Fig. 8). While the RNAi pathway remained highly active in the LRV-infected *L. guyanensis*, its activity as assayed with LUC or GFP reporters was attenuated ∼10-fold relative to that seen in virus-free *L. braziliensis* (Fig. 8A). Although tools for the introduction of LRV are not well-developed, one line of *L. guyanensis* has been described which was cured of LRV [38]. Notably the efficiency of RNAi in the virus free line was similar to that of the LRV1-4 containing line (Fig. 8A), showing that the attenuated RNAi response did not require the continued presence of virus. This implies that attenuation occurred through a down-regulation of the cellular RNAi pathway occurred in the LRV-bearing *L. guyanensis*. If a similar process occurred in the evolutionary lineage leading to extant RNAi-deficient *Leishmania* species, it could in turn have facilitated a later transition to a complete loss of RNAi activity. Future development of methods for more readily introducing and curing LRV infections will permit further tests of these hypotheses, as will the advent of RNAi-deficient lines of *Leishmania braziliensis* and other *Viannia* species. However, the data already in hand are consistent with the possibility of a biologically relevant interplay between parasite RNAi pathways and viral infection during evolution, as seen in viral infections of metazoans.

### Increased genome plasticity

A second selective force arises from consideration of the impact of genome plasticity in *Leishmania*. The ability of mobile elements to produce mutations and genomic rearrangements are well known, and in trypanosomes and other eukaryotes RNAi pathways may help protect against such events [42]–[44]. Importantly, the RNAi-competent *L. braziliensis* genome contains several classes of mobile elements, including retrotransposons, while RNAi-deficient *L. major* and *L. infantum* appear to lack active transposons [15]. While the forces leading to the loss of mobile elements are unknown, their departure could have freed the parasite from the need to maintain activities including RNAi which act to mitigate their effects.

Gene amplification is another important form of genomic plasticity in *Leishmania*, often occurring in the form of extra-chromosomal circular DNAs associated with drug resistance [45], [46]. In contrast, extra-chromosomal gene amplifications have not been seen in *T. brucei*, a difference potentially attributable to its active RNAi pathway [11], [13] since circular amplicons tend to be transcribed from both strands [47]. Consistent with this model, extrachromosomal gene amplifications are uncommon in RNAi-proficient *L. braziliensis* [48], and we found that transfections with a variety of circular DNAs were generally unsuccessful, causing us to rely exclusively on integrative constructs in this work. This does not imply that episomal circular DNAs will never arise in RNAi-proficient species; but when found, their transcription will be subject to RNAi effects and/or they will contain *cis*-acting elements that confer a high degree of strand specificity [49]. These requirements might act to constrain the emergence of episomal elements in RNAi-proficient species.

Thus the loss of RNAi could be seen as ‘freeing’ the genome of RNAi-deficient *Leishmania* from several constraints limiting genome plasticity. In this regards, loss of RNAi may be viewed as ‘mutator’ phenotype, similar to the ‘ARMed’ phenotype described recently in the malaria parasite *Plasmodium falciparum* or the high mutability phenotypes associated with elevated bacterial virulence in humans [50], [51].

### Phenotypic selection

Lastly, loss of RNAi may have been selected directly through effects on *Leishmania* virulence during evolution. The RNAi machinery affects gene expression at multiple levels, and its loss could lead to profound changes in parasite biology that could alter parasite virulence. Such direct alterations in gene expression may act in concert with the genomic alterations described above. The *Leishmania* subgenus *Viannia* is an early diverging clade within the genus [52], and these species exhibit a number of distinct features including the nature of the immune response in the mammalian host, the composition of their surface glycocalyx, and their behavior within the sand fly vector [8], [53]. Any such systematic differences between the RNAi-proficient *Viannia* subgenus and the RNAi-null *Leishmania* species groups could potentially reflect changes associated gene expression mediated by the RNAi pathway.

### Could RNAi be engineered into RNAi-deficient *Leishmania*?

Our findings provoke the question of whether the RNAi machinery could be transplanted from *L. braziliensis* into its close RNAi-deficient relatives. This would be useful given the extensive previous work on species such as *L. major* and *L. donovani*, as well as providing a tool for understanding the RNAi machinery. This feat was recently accomplished in *Saccharomyces cerevisiae*, which required only the introduction of Argonaute and Dicer from the closely related species *S. castellii* [33]. However, reintroduction of RNAi in *L. major* or *L. donovani* may require restoration of a more extensive suite of genes. While only three RNAi genes have been confirmed in trypanosomatids (an Argonaute and two Dicers) [9], [10], [17], preliminary data suggest a requirement for at least two additional genes (E. Ullu and C. Tschudi; unpublished data). Importantly, all 5 genes are absent in the genomes available for RNAi-deficient *Leishmania* species. In other eukaryotes the RNAi machinery includes as many as 9 proteins or more [15], [31], [54]. Another obstacle may be the tendency of RNAi-deficient species such as *L. major* to transcribe the antisense chromosomal strand at low levels [55], as well as to synthesize antisense transcripts [56], [57]. This suggests the possibility that introduction of an active RNAi pathway into *L. major* could be lethal [11], [58]. Thus re-introduction of RNAi into RNAi-deficient *Leishmania* species will be a challenging task; nonetheless, efforts to introduce this suite of genes from RNAi proficient *L. braziliensis* are underway.

In summary, we have shown that the RNAi pathway is functional in *Leishmania braziliensis*. These data provide some optimism for the application of RNAi approaches as a tool for the study of these predominantly asexual organisms, by forward and reverse genetic approaches. While less experimentally developed, *L. braziliensis* has the potential to emerge as an attractive model, and the advent of RNAi-based tools should provide a further stimulus for this effort. In the long term, delivery of siRNAs targeting essential parasite genes may prove an effective route to chemotherapeutic treatment of RNAi-proficient *Leishmania*. Lastly, the *Leishmania* provide an attractive system for testing hypotheses about forces leading to the evolutionary loss of RNAi, including the role of viral pressure, changes in genome plasticity, and virulence. As drug resistance mediated by gene amplification is one manifestation of gene plasticity, these findings have practical implications to parasite chemotherapy.

## Materials and Methods

### Northern blotting

RNA extraction procedures and Northern analyses were carried out as described [16]. The 5′UTR of *L. braziliensis* α-tubulin mRNA plus the first 317 nt of the ORF were PCR-amplified from genomic DNA and inserted between the *Hind*III and *Xba*I sites of plasmid vector pPD19.36, which contains two opposing T7 RNA Polymerase promoters [59]. The synthesis of dsRNA was according to Ngo et al. [21]. The same DNA was used as a probe in the α-tubulin Northern. PCR products of GFP+ or GFP65 ORFs were used as probes for the GFP Northerns. A portion (nt 3160 to nt 4482) of the *L. braziliensis SLACS* (LbrM08_−_V2.0700) was PCR-amplified with primers (LBSLACS1399F: 5′-GCCAGAGAGGTGGTGAGGGTG and LBSLACSORFa-R: 5′-GAGCTCGAGAAAGGTCCACCACCCCGA) from M2903 genomic DNA and TA cloned to generate a sense radiolabeled RNA probe for Northern analysis of small RNAs. For *LPG2* (LbrM20_V2.2700) the probe was a PCR fragment (nt 1 to nt 411) amplified with primers SMB3219 and SMB3220 (Table S1).

### RNA preparation and quantitative real-time PCR (qRT-PCR)

*Leishmania* total RNA was isolated using the Trizol reagent (Invitrogen), treated with DNAse and purified using MEGAclear columns (Ambion). Reverse transcription (RT) was performed according to the manufacture instructions using Superscript III First-Strand reverse transcriptase (Invitrogen) in a 20 µl reaction containing 1µg purified RNA. Controls containing the same amount of RNA but lacking reverse transcriptase or template were used to rule out DNA or other contamination. For test RNAs, primers were designed to amplify ∼100 bp amplicons within the target ORF but outside of the stem-fragment, and tested using *L. braziliensis* gDNA. PCRs were performed using the SYBR Green (Applied Biosystems) and the ABI PRISM 7000 Sequence Detection System instrument (Applied Biosystems). PCR amplifications were performed as follows: 50°C for 2 min and 95°C for 10 sec then followed by 40 cycles of 95°C for 15 sec, 60°C for 1min. The generation of specific PCR products was confirmed by melting curve analysis and agarose gel electrophoresis. Each primer set was individually tested for four *StL* transfectants (2 for *StL*-F and 2 for *StL*-R; except 4 for *LPG3*-*StL*-F). All samples were performed in triplicate. Control samples of H_2_O were included in each experiment. Amplification of SSU rRNA was used as internal control to normalize the parallel reaction of target amplicons.

### *Leishmania* strains

*L. braziliensis* M2903 (MHOM/BR/75/M2903), *L. guyanensis* M4147 (MHOM/BR/75/M4147) and *L. panamensis* WR120 (MHOM/PA/74/WR120) were obtained from Diane McMahon-Pratt (Yale University), *L. braziliensis* strain M2904 from Angela Cruz (U. Sao Paulo Riberao Preto), *L. tarentolae* strain TarII was obtained from M. Ouellette and B. Papadopoulou (U. Laval), *L. mexicana* (MNYZ/BZ/62/M379) from David Russell (Cornell University), and *Crithidia fasciculata* Cf-C1 from Larry Simpson (UCLA). The LRV-bearing strain of *L. guyanensis* M4147 (MHOM/BR/75/M4147) and a virus free derivative M4147/pX63-HYG [38] were obtained from Jean L. Patterson (Southwest Foundation for Biomedical Research, San Antonio, Texas). The identities of all *Viannia* strains used were confirmed by partial and/or complete sequencing of the *AGO1* or other genes (not shown).

*Viannia* specie*s* were grown in freshly prepared Schneider's Insect Medium (Sigma-Aldrich Cat. No. S9895) supplemented with 10% heat-inactivated fetal bovine serum, 2 mM L-glutamine, 500 units penicillin/ml and 50 µg/ ml^−^ streptomycin (Gibco Cat No. 5070). Other *Leishmania* and *Crithidia* were propagated in M199 medium supplemented with 10% heat-inactivated fetal bovine serum, hemin, adenine, biopterin and biotin [60].

### Transient and stable transfection

For each transfection, 10 ml of log phase *L. braziliensis* were resuspended in 100 µl human T-cell Nucleofector solution (Amaxa Cat No. VPA-1002) mixed with 5 µl of 4 µg/ µl of α-tubulin dsRNA or control dsRNA and subjected to nucleofection with an Amaxa Nucleofector with program U-033 using the kit's cuvette. The transfection mixture was transferred immediately to 10 ml of complete medium and kept in 28°C for 3 hrs. RNA from 9 ml cells was taken for Northern blot analysis with an α-tubulin hybridization probe.

Stable transfections were performed using the high voltage (1400V) procedure described previously [11]. Following electroporation organisms were grown in drug-free media overnight, and then plated on semisolid media [60] to obtain clonal lines. For selections using the *SAT* marker, parasites were plated on 50–100 µg/ml nourseothricin (clonNAT, Werner BioAgents, Germany), and with the *PHLEO* marker, parasites were plated on 0.2–2 µg/ml phleomycin (Sigma). After colonies emerged (typically <2 weeks) they were recovered and grown to stationary phase in 1 ml media, and passaged thereafter in 10 and 0.1 µg /ml nourseothricin and phleomycin, respectively. The plating efficiency of untransfected *L. braziliensis* M2903 ranged from 60–95% and the transfection efficiency from 50–220 colonies / 20 µg DNA.

### *AGO1* sequencing

The generation of whole genome shotgun sequence data from *Crithidia fasciculata* strain Cf-C1 by 454 sequencing technology will be described fully elsewhere. Blast searches using *L. braziliensis AGO1* were used to identify homologous sequences, which were then assembled manually into several large contigs. PCR primers were designed to amplify missing gaps, and the 5′ end of the mRNA was obtained by RT-PCR using a forward miniexon primer (CFSLB 5′-AAGTATCAGTTTCTGTACTTTATTG) and reverse *CfAGO1* specific primer (SMB2895: 5′-AAGCAGTTCGTCTCCACCGTCACCTG). Then a nested PCR was done with CfSLB and CfAGO1 primers (SMB 2894: 5′- GTGATGCCGCCCTCCTCGCGGTCACG). The PCR products were TA cloned and sequenced. The *CfAGO1* sequence was deposited in GenBank (EU714010). We noted a polymorphism in the *CfAGO1* sequence, introducing a stop codon yielding a truncated protein terminating after amino acid 198. The consequences of this polymorphism (if any) have not been investigated further. The sequence of the *L. guyanensis* M4147 *AGO1* ORF was determined by direct sequencing of the PCR amplicon obtained with primers B2468 (5′-ATGTTGGCGCTAAACGCAGGTTC) and B2469 (5′- CTACAGGTAGTGCATCGTGGGGC), and deposited in GenBank (accession number FJ234150).

### Detection of LRV virus

RT-PCR reactions were performed as described above, with two sets of primers to detect LRV viruses described previously [38] (set 1, primers SMB2472/2473 and set 2, primers SMB3850/3851 (Table S1).

### Constructs

The constructs used in this work are derivatives of pIR1SAT (B3541) [11] or pIR1PHLEO (B4054, this work), which have two expression sites (*Xba*I/*Sma*I, site a, and *Bgl*II, site b). High fidelity thermostable polymerases such as recombinant *Pfu* DNA polymerase (Stratagene) were used for PCR, and constructs were confirmed by restriction mapping and sequencing of all relevant regions. Unless otherwise indicated, all constructs were digested with *Swa*I and the linear *SSU*-targeting fragment purified for subsequent transfection by electroporation.

### Reporters

pIR1PHLEO (B4054) was created by replacing the *SAT* marker of pIR1SAT with the *PHLEO* marker (M. Cunningham, unpublished data). pIR1PHLEO-GFP+(a) (B5793), pIR1PHLEO-GFP65(a) (B5779) and pIR1-GFP65*(a) (B5959) were constructed by generating ORF cassettes of the respective genes and inserting into the *Xba*I (a) site. The GFP+ ORF was taken from pXG-GFP+ (B2799), GFP65 from pXG-GFP65 (B2355), and GFP65* was obtained by site-specific mutagenesis of pIR1PHLEO-GFP65 (QuickChange Multi Site-Directed Mutagenesis, Stratagene), changing nt 193 from T to A, resulting in a S65T mutation. A luciferase (*LUC*) ORF was amplified using pGL3-basic (Promega) as template, with primers adding flanking *Bgl*II sites, and a CCACC initiation sequence preceding the initiation codon. The modified *LUC* ORF was inserted into pGEM-T (Promega) yielding pGEM-Luciferase (B6033); the *LUC* ORF was then extracted by *BglII* digestion and inserted into the *Bgl*II site of B5959 to create pIR1PHLEO-GFP65*(a)-LUC(b) (B6034).

### Stem-Loop (StL) constructs

pIR1SAT-GFP65-StL(b) (B4733) was described previously [11]. For other StL constructs, we assembled a stem-loop consisting of the target gene sequences in inverted orientation, separated by a PEX11-MYC(3) loop/stuffer fragment used previously in pIRGFP Stem-Loop (B4733), and inserted this into either the ‘a’ or ‘b’ expression sites of pIR1SAT. In these constructs the ‘stem’ sequences were organized either in divergent or convergent orientations (DIV or CONV) relative to the target gene sequence, and the stuffer fragment similarly could be in a ‘sense’ or ‘antisense’ orientation relative to PEX11 (F or R). The specific target genes and regions studied included *LPG1* (LbrM25_V2.0010, nt 11–592); *LPG2* (LbrM20_V2.2700, nt 411–1021); *LPG3* (LbrM29_V2.0780, nt 1657–2236); *HGPRT* (LbrM21_V2.0990, nt 127–626); α-tubulin (LbrM13_V2.0190, nt 736–1309); β-tubulin (LbrM33_V2.0930, nt 470–1004); *PFR1* (LbrM31_V2.0160, nt 900–1593); *PFR2* (LbrM16_V2.1480, nt 951–1644), *AGO1* (LbrM11_V2.0360, nt 247–1070) and *LUC* (LUC+ from Promega pGL3-Basic, nt 281–788). These steps yielded constructs pIR1SAT-*LPG1-StL* (b,DIV,R)(B6128), pIR1SAT-*LPG1-StL* (b,DIV,F) (6132), pIR1SAT-*LPG2-StL*(b,DIV,R) (B6137), pIR1SAT-*LPG2-StL*(b,DIV,F) (B6138), pIR1SAT-*LPG3-StL*(b,DIV,F) (B6140), pIR1SAT-*HGPRT-StL*(b,DIV,F) (B6136), pIR1SAT-*HGPRT-StL*(b,DIV,R) (B6135), pIR1SAT-*PFR1-StL*(b,DIV,F) (B6294), pIR1SAT-*PFR2-StL*(b,DIV,F) (B6282), pIR1SAT-*αTub-StL*(b,DIV,F) (B6283), pIR1SAT-*βTub-StL*(b,DIV,F) (B6295) and pIR1SAT-*LUC-StL*(b,CONV,F) (B6185), or pIR1SAT*-LUC-StL*(b,DIV,F) (B6190).

### LUC self reporter (LUC SR) and RNAi of *AGO1*

A single construct enabling tests of RNAi activity was generated by inserting the LUC ORF into the ‘b’ site and a *LUC* Stem-Loop into the ‘a’ site of a modified pIR vector (pIR2SAT-LUC-StL(a)-LUC(b) (B6386). This construct is referred to as the ‘LUC RNAi self reporter’ or ‘LUC SR’. For RNAi studies of *AGO1*, an analogous construct was made with a HYG marker (pIR2HYG-LUC-StL(a)-LUC(b), strain B6447). A pIR1SAT-LbrAGO1-StL(b) construct was used for RNAi tests (B6524).

### LPG, Western blots and GFP flow cytometry

Western blots were performed as described elsewhere using anti-GFP (Abcam Cat No. 6556, 1∶2500) or anti-*L. donovani* HGPRT antiserum (1∶5000; J. Boitz and B. Ullman, Oregon Health Sciences University) as the primary antibody, and detected using goat anti-rabbit IgG as the secondary antibody (1∶10000, Jackson ImmunoResearch Laboratories, Inc. catalog number 111-035-003). Parasites expressing GFPs were analyzed using a Becton-Dickenson FACS Calibur, using fluoroscein excitation/emission parameters. LPG was purified and quantitated from *L. braziliensis* lines grown in logarithmic phase (4–5×10^6^ cells/ml) as described [61]. Purified LPG was subjected to western blotting with antisera CA7AE which recognizes the Gal(β1,4)Man(α1-P) repeat units of the *L. braziliensis* LPG [62].

### Luciferase assay

10^6^ logarithmic phase promastigotes were suspended in 200 µl media containing 30 µg/ ml of luciferin (Biosynth AG) and added to a 96-well plate (Black plate, Corning Incorporated, NY, U.S.A.). After 10 min incubation, the plate was imaged using a Xenogen IVIS photoimager (Caliper LifeSciences), and luciferase activity quantitated as photons/sec (p/s).

### Transmission electron microscopy

Promastigotes were fixed in 2% paraformaldehyde/2.5% glutaraldehyde (Polysciences Inc., Warrington, PA) in 100 mM phosphate buffer, pH 7.2 for 1 hr at room temperature. Samples were washed in phosphate buffer and postfixed in 1% osmium tetroxide (Polysciences Inc., Warrington, PA) for 1 hr. Samples were then rinsed extensively in water prior to en bloc staining with 1% aqueous uranyl acetate (Ted Pella Inc., Redding, CA) for 1 hr. Following several rinses in water, samples were dehydrated in a graded series of ethanol solutions and embedded in Eponate 12 resin (Ted Pella Inc.). Sections of 95 nm were cut with a Leica Ultracut UCT ultramicrotome (Leica Microsystems Inc., Bannockburn, IL), stained with uranyl acetate and lead citrate, and viewed on a JEOL 1200 EX transmission electron microscope (JEOL USA Inc., Peabody, MA).

## Supporting Information

Table S1 Primers used for qRT-PCR.
(0.05 MB DOC)

Figure S1 SLACS-derived siRNAs. siRNA analysis of RNAs from *L. braziliensis* M2903 promastigotes and *Trypanosoma brucei* procyclics. This Northern blot was probed with a *L. braziliensis* SLACS probe and the autoradiogram is shown. Trypanosome SLACs differs greatly in sequence from that of *L. braziliensis* and as expected no siRNA hybridization is evident. Size standards are in the left track.
(0.47 MB TIF)

Figure S2 GFP Reporters ORF nucleotide alignment. An alignment of the AT-rich GFP65 ORF and GC-rich GFP+ nucleotide sequences is shown. The T→A mutation in GFP65* (S65T in the protein) is indicated. Regions of identity are boxed.
(2.87 MB TIF)

Figure S3 Tests of RNAi in *L. braziliensis* M2903 with lines bearing a Luciferase reporter subsequently transfected with LUC-StL. Luciferase activity (photons/sec or p/s) was measured as described in the Methods. Control parasites were WT *L. braziliensis* M2903 (Lb WT) and L. braziliensis M2903 expressing luciferase (Lb+LUC; SSU:PHLEO:GFP65*(a)-*LUC*(b)). Test transfectants of Lb+LUC additionally expressed the LUC-StL in a convergent (*SSU:SAT:LUC-StL(b-CONV)*; **Panel A**) or divergent orientation (*SSU:SAT:LUC-StL(b-DIV)*; **Panel B**). GFP expression varied less than 10% amongst experimental samples.
(0.33 MB TIF)

Figure S4 GFP siRNAs in *Crithidia. Crithidia fasciculata* clone Cf-C1 was electroporated with the targeting fragment from pIR1SAT-HYG(a)-GFP(65)-StL(b), yielding *SSU:SAT-HYG-GFP(65)-StL* transfectants. These were confirmed by PCR tests for the marker and presence of the inverted *GFP65* repeats, and RNA was isolated and subjected to Northern blotting for siRNAs using a *GFP65* probe.
(0.94 MB TIF)
