# Supplementary material for: Retention and Loss of RNA Interference Pathways in Trypanosomatid Protozoans
Source: PLoS Pathog. 2010 Oct 28;6(10):e1001161. doi: 10.1371/journal.ppat.1001161 (PMC2965760; doi:10.1371/journal.ppat.1001161)
Supplement: Table S1 — Primers used for qRT-PCR. (0.05 MB DOC) [file ppat.1001161.s001.doc]

**Table S1.**  Primers used in qRT-PCR.

| Gene (amplicon location) | Primer sequence(5’🡪 3’) |
| --- | --- |
| *LPG1* (nt 601-700) | F (3217): GAACGTATTCGCCGCCTCGACG |
|  | R (3256): TTGGGAGGGTGGAGCTGCGGAA |
| *LPG1* (nt 1191-1290) | F (3257): ACTCGACACAGAGAGGCAAAGG |
|  | R (3218): TTAGAAGCGCAGGAGGCTCGTG |
| *LPG2* (nt 311-410) | F (3333): CGATTATCAAAAACTTGGCTGT |
|  | R(3220): ATCAACGCAAACGACACGTACA |
| *LPG2* (nt 1-100) | F (3219): ATGAACCACTCCCACATTGTGA |
|  | R (3332): TGTTCATGATCAGCTTGTTCAC |
| *LPG3* (nt 1529-1650) | F (3456): GCATGAAGAAGGGACAGAAAGG |
|  | R (3222): GTCCGTCATGAAGATCACCTCA |
| *LPG3* (nt 901-1000) | F (3221): ACCCGCCAGATCGGTAACGTGA |
|  | R (3455): CCTTGAAGTGGCTGAAGTATAA |
| *HGPRT* (nt 1-100) | F (3324): ATGAGCGGCTGGACCAAGTCCC |
|  | R (3325): ACACCTGCTCCTGCGTGATGAC |
| SSU rRNA | F (3180): TCTAGGCTACCGTTTCGGCTT |
|  | R (3181): CACACACCGAACCGAAGTTG |
| LRV1-4 (set 1) | F (2472): GCATACCGTTTTGAGTGGAC  R (2473): GTTTCAATCATTGGCTGACA |
| LRV1-4 (set 2) | F (3850): TGTTACTTACCCTACGACTC  R (3851): TGTGTAAGAAGTCAACT |
|  |  |
|  |  |
