# Supplementary figures and images for: Retention and Loss of RNA Interference Pathways in Trypanosomatid Protozoans
Source: PLoS Pathog. 2010 Oct 28;6(10):e1001161. doi: 10.1371/journal.ppat.1001161 (PMC2965760; doi:10.1371/journal.ppat.1001161)

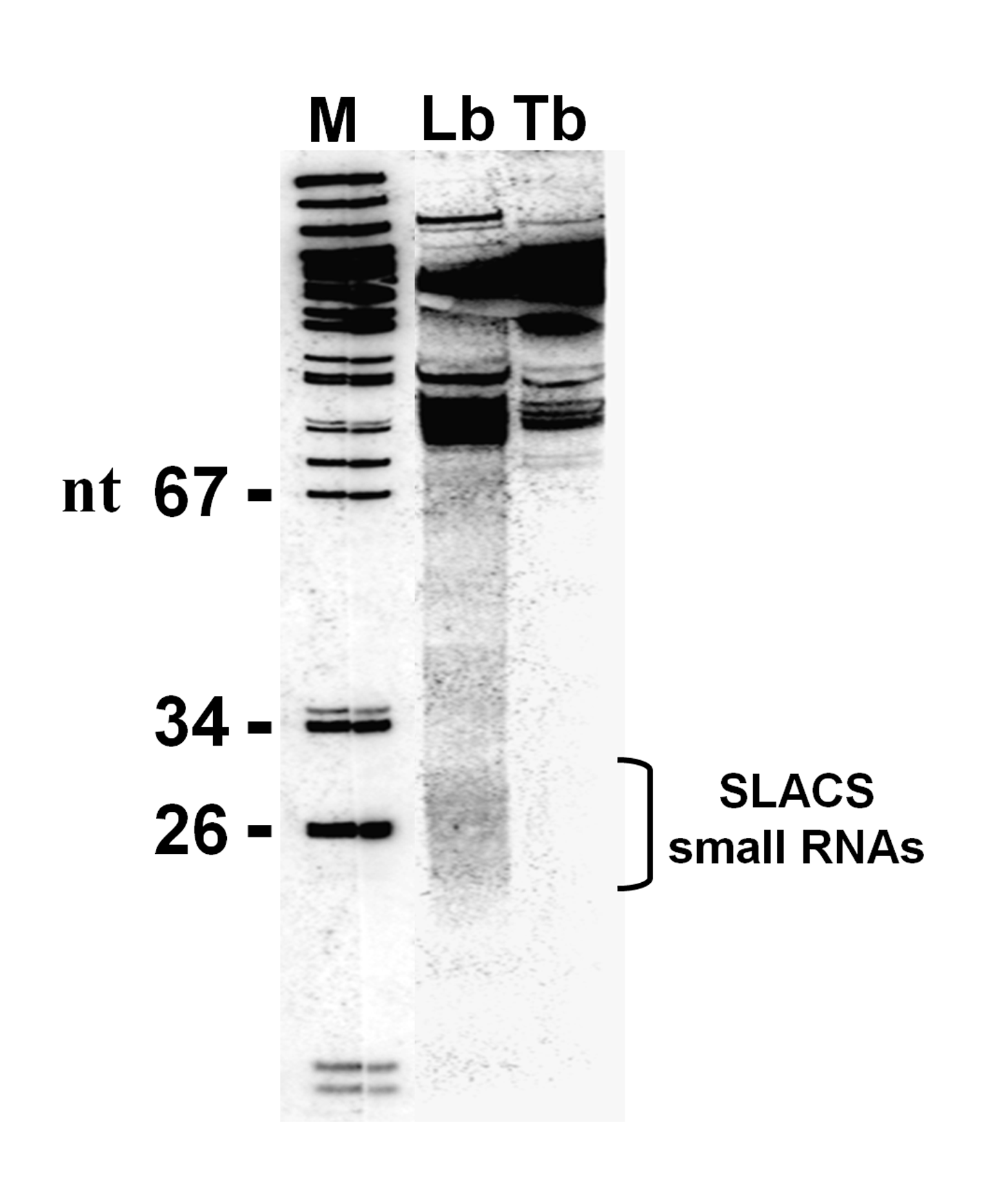

Supplement: Figure S1 — SLACS-derived siRNAs. siRNA analysis of RNAs from L. braziliensis M2903 promastigotes and Trypanosoma brucei procyclics. This Northern blot was probed with a L. braziliensis SLACS probe and the autoradiogram is shown. Trypanosome SLACs differs greatly in sequence from that of L. braziliensis and as expected no siRNA hybridization is evident. Size standards are in the left track. (0.47 MB TIF) [file ppat.1001161.s002.tif]

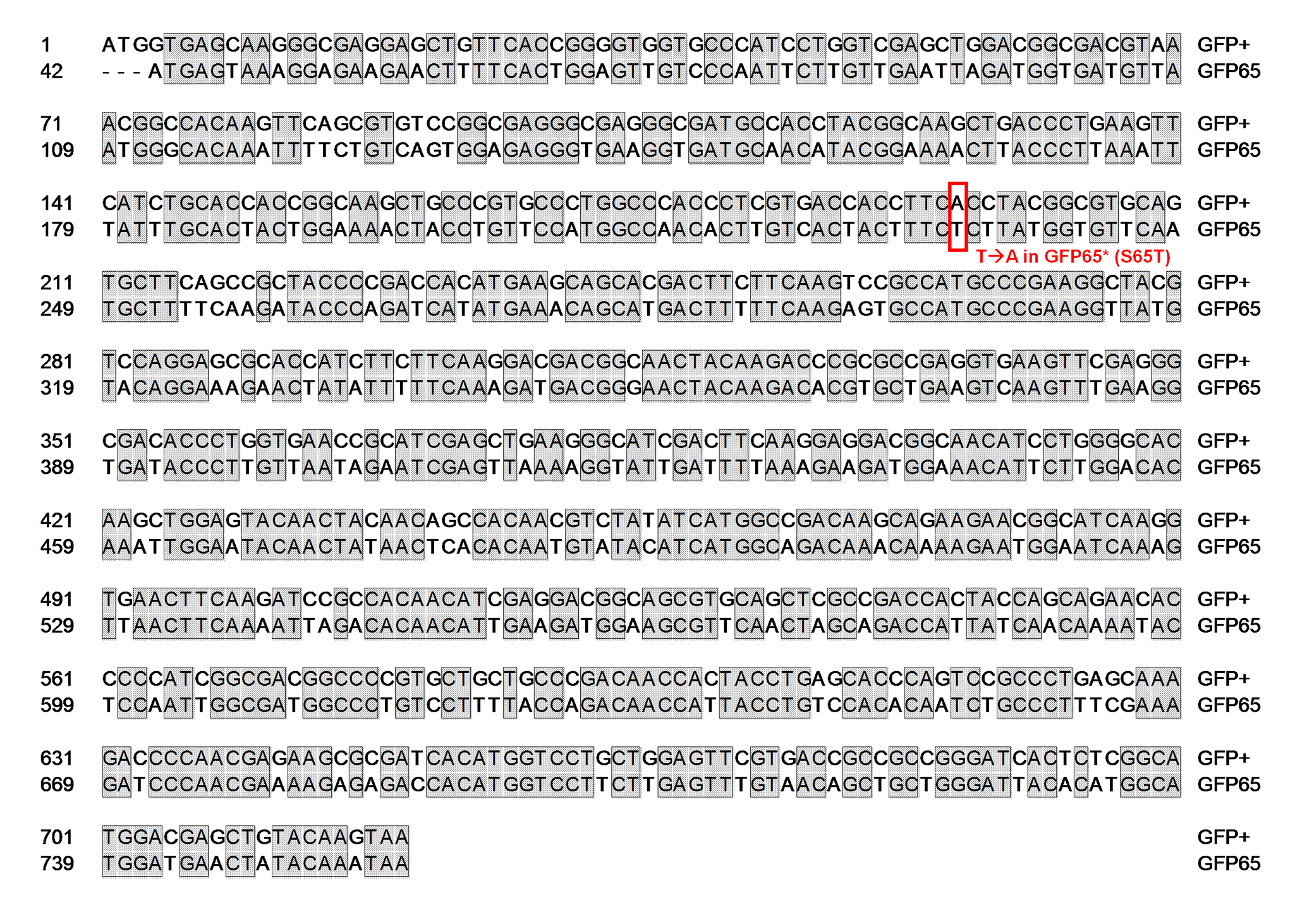

Supplement: Figure S2 — GFP Reporters ORF nucleotide alignment. An alignment of the AT-rich GFP65 ORF and GC-rich GFP+ nucleotide sequences is shown. The T→A mutation in GFP65* (S65T in the protein) is indicated. Regions of identity are boxed. (2.87 MB TIF) [file ppat.1001161.s003.tif]

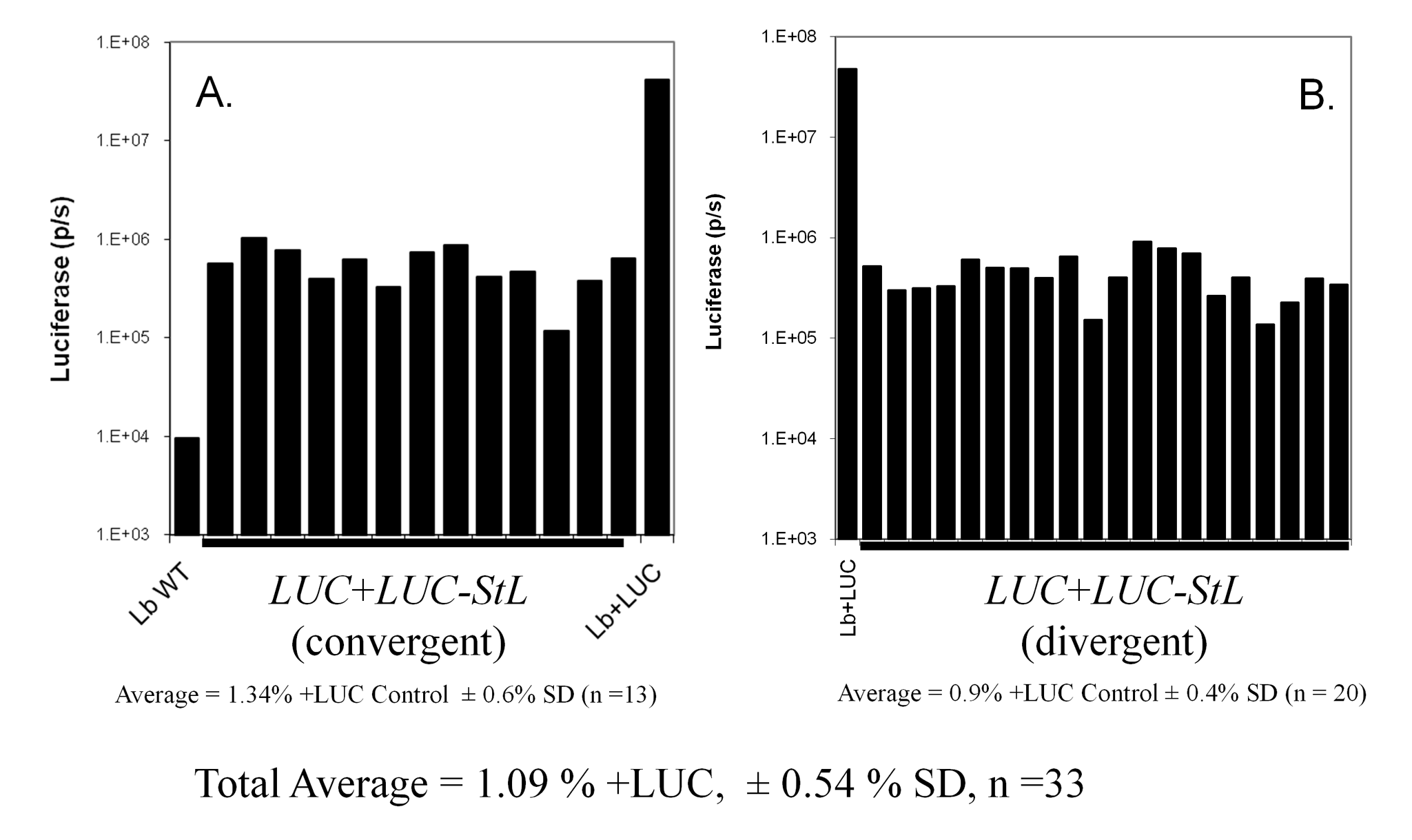

Supplement: Figure S3 — Tests of RNAi in L. braziliensis M2903 with lines bearing a Luciferase reporter subsequently transfected with LUC-StL. Luciferase activity (photons/sec or p/s) was measured as described in the Methods. Control parasites were WT L. braziliensis M2903 (Lb WT) and L. braziliensis M2903 expressing luciferase (Lb+LUC; SSU:PHLEO:GFP65*(a)-LUC(b)). Test transfectants of Lb+LUC additionally expressed the LUC-StL in a convergent (SSU:SAT:LUC-StL(b-CONV); Panel A) or divergent orientation (SSU:SAT:LUC-StL(b-DIV); Panel B). GFP expression varied less than 10% amongst experimental samples. (0.33 MB TIF) [file ppat.1001161.s004.tif]

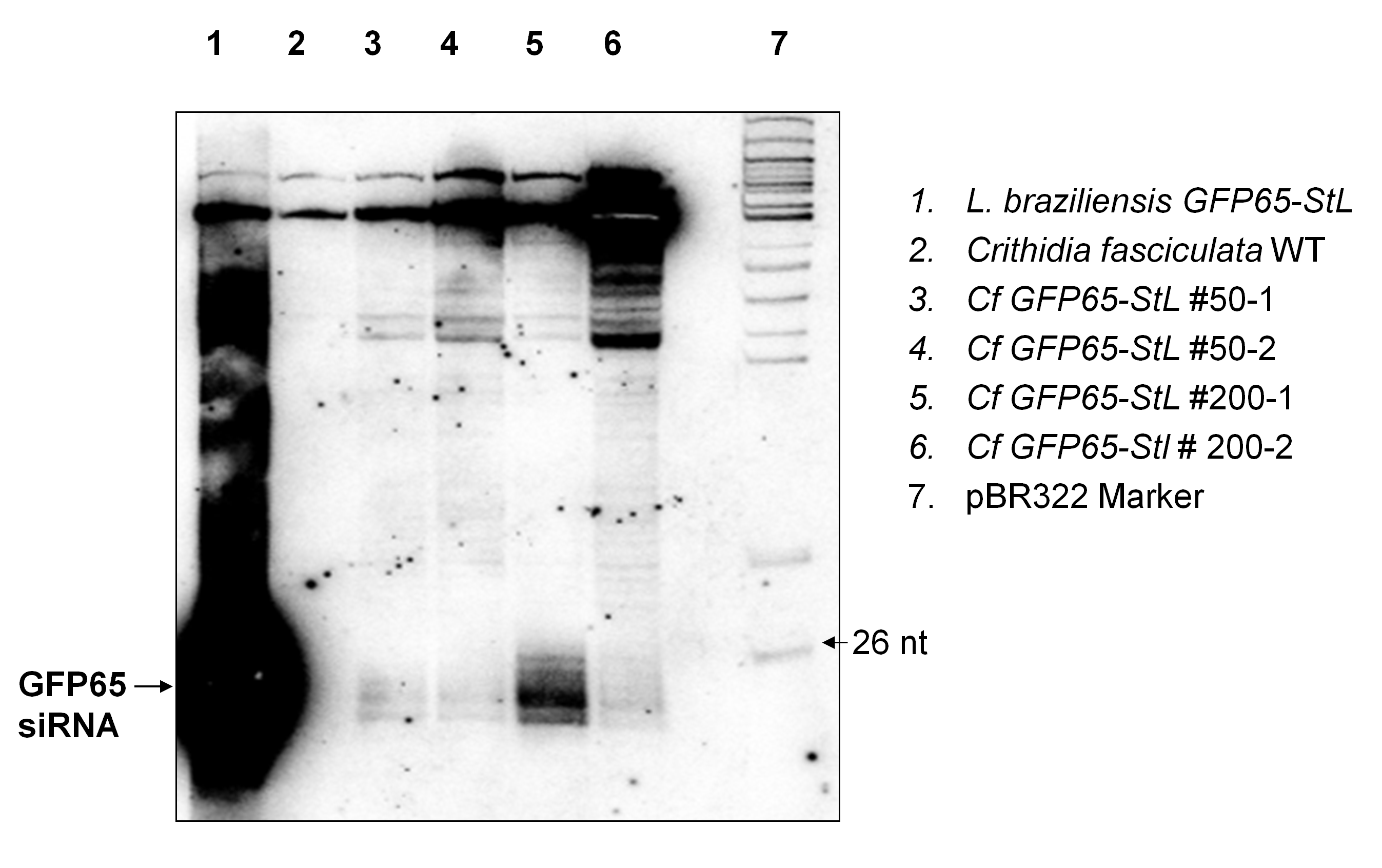

Supplement: Figure S4 — GFP siRNAs in Crithidia. Crithidia fasciculata clone Cf-C1 was electroporated with the targeting fragment from pIR1SAT-HYG(a)-GFP(65)-StL(b), yielding SSU:SAT-HYG-GFP(65)-StL transfectants. These were confirmed by PCR tests for the marker and presence of the inverted GFP65 repeats, and RNA was isolated and subjected to Northern blotting for siRNAs using a GFP65 probe. (0.94 MB TIF) [file ppat.1001161.s005.tif]
